# The association of overhydration with megafistulas in hemodialysis patients

**DOI:** 10.1080/0886022X.2019.1614954

**Published:** 2019-06-04

**Authors:** Mihály Tapolyai, Mária Faludi, Klára Berta, Melinda Forró, Lajos Zsom, Ákos G. Pethő, László Rosivall, Tibor Fülöp

**Affiliations:** a Semmelweis University, Budapest, Hungary;; b Hemodialysis Unit, Fresenius Medical Care, Budapest, Hungary;; c Medical Services, Ralph H. Johnson VA Medical Center, Charleston, SC, USA;; d Hemodialysis Unit, Fresenius Medical Care Hungary, Hatvan, Hungary;; e Hemodialysis Unit, Fresenius Medical Care Hungary, Cegléd, Hungary;; f 1st Department of Internal Medicine, Faculty of Medicine, Semmelweis University, Budapest, Hungary;; g Department of Pathophysiology, International Nephrology Research and Training Center, Semmelweis University, Budapest, Hungary;; h Department of Medicine, Division of Nephrology, Medical University of South Carolina, Charleston, SC, USA

**Keywords:** Bioimpedance spectroscopy, blood pressure, body composition, end-stage renal disease, megafistula, volume overload

## Abstract

**Objectives:** Diffuse enlargements of arteriovenous dialysis fistulas customarily attributed to either excessive arterial inflow or central outflow stenosis. The relationship between volume status and clinically enlarged (arteriovenous) fistula (CEF) formation in end-stage renal disease (ESRD) patients is not well understood.

**Methods:** We assessed the pre-dialysis bioimpedance spectroscopy-measured percentage of overhydration (OH%) in 13 prevalent dialysis patients with CEF development and negative angiography and compared the results with those of 52 control dialysis patients (CONTR). All patients were prevalent ESRD patients receiving thrice-weekly maintenance hemodiafiltration at an academic outpatient dialysis unit.

**Results:** 10/13 CEF patients had OH% ≥15% as compared to 20/52 control patients (Chi square *p*: .02). The degree of OH% was 20.2 ± 7.4% among the CEF vs. 14.4 ± 7.1% in the control group (Student’s *t*-test *p*: .01), representing 4.2 ± 3.2 vs. 2.8 ± 1.6 L of excess fluid pre-dialysis (*p*: .03). Patients with CEF development took an average of 1.7 ± 1.4 vs. 0.8 ± 0.8 (*p*: .002) antihypertensive medications compared to the CONTR patients, yet their blood pressure was higher: 156/91 vs. 141/78 mmHg (systolic/diastolic *p*: .03<.0001). We found no difference in fistula vintage, body mass index, age, diabetes status, or diuretic use. The odds ratio of having a CEF in patients with ≥15% OH status was 5.3 (95% CI: 1.3–21.7; *p*: .01), the Number Needed to Harm with overhydration was 4.

**Conclusions:** There is an association between bioimpedance spectroscopy-measured overhydrated clinical state and the presence of CEF; either as an increased volume capacitance or as a potential cause.

## Introduction and background

For decades, arterio-venous fistulas (AVFs) have been deemed the primary and desired dialysis access for hemodialysis [1], with the successful creation and maintenance of AVFs remaining Achilles’ heel of hemodialysis. Most dialysis fistulas fail by stenosis and clotting, representing the principal cause of access loss in hemodialyzed patients, more commonly occurring in diabetics [2] or those with higher hemoglobin [3]. Early failure of arteriovenous dialysis access occurs at a rate of about 17.5% [4] to 23% [5] and the overall one-year primary patency rate is about 60%. Most efforts to maintain and salvage fistulas have focused so far on the preservation of blood flow in the arterial inflow or venous outflow tracts.

Another, albeit much less common mechanism of hemodialysis access failure is the development of fistulas with excessively high flow and large diameters, also known as megafistulas [6], most commonly originating from the brachio-cephalic location. Established risk factors for megafistulas are wide arterio-venous anastomoses with large blood flow rates or a relative narrowing of the draining vein in the venous circulation. Megafistula formation is most commonly recognized by an excessive blood flow and clinically confirmed by the Nicoladoni-Branham maneuver. The suggested criteria include an increased blood flow greater than 2.2 L/min, increased cardiac output and index, increased (>20%) cardio-pulmonary recirculation and hypertrophied feeding artery [6,7].

Another working definition of megafistula simply refers to the ultrasonically measured blood flow rate within the fistula. ‘Although it is uncertain as to why some patients develop a megafistula, altered hoop stress (circumferential stress) almost certainly plays a role’ [8]. The exact relationship between volume status and enlarged fistula formation is, nonetheless, currently insufficiently understood. Objective assessment of volume status in end-stage renal disease (ESRD) remains challenging and persisting chronic volume overload may contribute to poor BP control [9,10], vascular stiffness [10,11], and decreased survival [12–15]. Bioimpedance monitoring is an emerging gold standard methodology to assess not only body composition [16–18] but to assess the exact degree of volume status in dialysis patients [14–16,19]. As the relationship between volume status, antihypertensive drug use status and an enlargement of AVF is not well understood, we investigated whether volume overload may represent an additional risk factor for these redundant vascular enlargements.

## Methods

This was a case-control study approved by the Ethics Committee of the Hungarian Health Ministry (equivalent for an Independent Review Board) (TUKEB 3032-1/2015/EKU) and Fresenius Medical Care as the dialysis provider in Hungary. The study conformed to the Helsinki Declaration as developed by the World Medical Association. The data used for the study were obtained during routine care of maintenance dialysis at the Fresenius Medical Care facilities, within the premises of and affiliated with Semmelweis University in Budapest, Hungary. All patients received thrice weekly maintenance hemodiafiltration using a post-dilution fluid replacement method, aiming at a single-session *Kt*/*V* of 1.4 and a replacement fluid volume of 21 L [20].

All of our patients routinely undergo bimonthly pre-dialysis fluid status evaluations at the designated medical care facility by using a multi-channel bioimpedance spectroscopy apparatus (BCM – Body Composition Monitor, Software version 3.2; Fresenius Medical Care, Bad Homburg, Germany) as part of our standard clinical practice. The etiology of the proband group’s ESRD was heterogeneous including PCKD (1), urological anomalies (1), nephrectomy because of renal cell carcinoma (2), presumably hypertension (1), diabetes (3), glomerulonephritis (2), and unknown. We have previously described the measurement method [16]; however, it shall be added that we measured the patients’ fluid compartments by positioning them flat on their backs and placing two conductive electrodes on their hands and ankles on the same side at the same time. With this method, we measured their total body water (TBW), extracellular water (ECW), intracellular water (ICW), and overhydration (OH) levels in liters and acquired the percentage (OH%) of the excess fluid that is greater than the anticipated ECW. The validation of the bioimpedance methods for measuring fluid spaces has been done historically with various isotopes (of potassium, bromide, hydrogen), tagged albumin and DEXA scans and reviewed earlier in details [21].

Our goal was to define pre-dialysis BCM-measured OH% in 13 prevalent chronic dialysis patients with CEF. The diagnosis of CEF was established on clinical exam, with excessive and torturous dilatations of arteriovenous fistula observed throughout its course in the extremity; these were typically easy to palpate and traceable to the shoulder. We did not have the means to measure blood flow in the fistulas; consequently, the diagnosis of ultrasonography-determined megafistula could not be made with certainty and the diagnosis of clinically enlarged AVF was made purely on clinical grounds, by the consensus of the nephrologists involved in the clinical care of the cohort. Due to this lack of diagnostic rigor, we refer to these fistulas as clinically enlarged fistulas (CEFs). All CEF subjects had negative angiographies performed earlier by a dedicated interventional radiologist and definitively ruled out for proximal outflow stenosis. We compared these patients with 52 consecutive control dialysis patients without enlarged fistula formation on clinical grounds but no further testing (Doppler ultrasound or angiography) was performed. We tabulated the patients’ demographic characteristics as well as their blood pressures and blood pressure medication numbers. For the comparison of continuous data, we utilized Student’s *t*-tests and for categorical data, we explored statistical significance with two-tailed Fisher’s exact test statistics.

## Results

**Figure 1.**
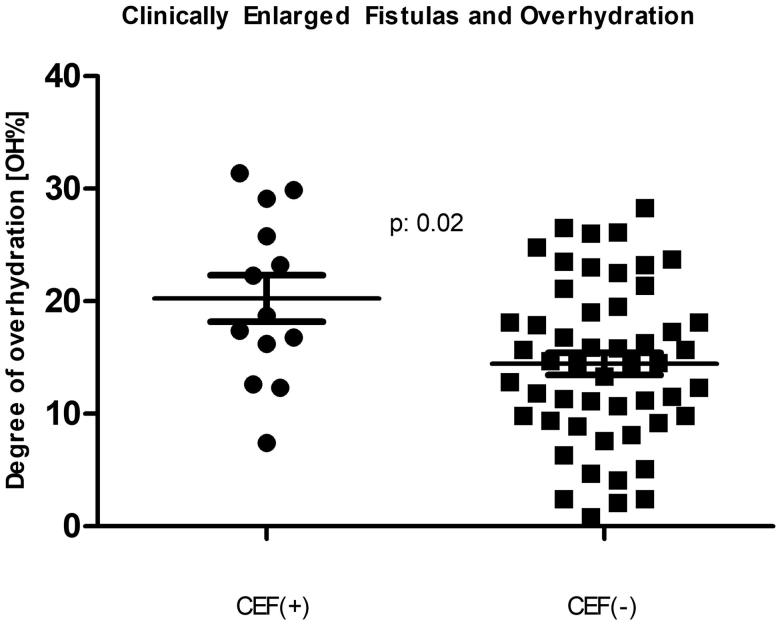
Comparison of those with and without a clinically enlarged arteriovenous fistula (CEF) with respect to the bioimpedance-measured fluid overhydration. The two populations’ percent-overhydration (*n*: 13 and *n*: 52) mean ± standard deviation is depicted by the horizontal lines.

The characteristics of the patients are depicted in Table 1. The CEF as well as the control patients’ data passed the normality test for the Gaussian distribution when tested by the D’Agostino and Pearson omnibus normality test, the Shapiro normality test, and the Kolmogorov–Smirnov test for normality. There was a statistically significant male predominance (84.6% vs. 51.9%; *p*: .03) among the subjects with CEFs, but they did not differ in age, dialysis vintage, prevalence of diabetes mellitus or even the amount of residual urine volume from controls. Importantly, however, the CEF patients had higher blood pressure, took more antihypertensive medications and were significantly more overhydrated (*p* < .01 for all). The degree of overhydration, when expressed in liters showed 1.4 L of excess extracellular fluid (Student’s *t*-test *p*: .03; Mann–Whitney *p*: .06) among the subjects with CEF. Moreover, when the degree of volume overload was expressed as a % of OH, the difference became striking (Student’s *t*-test *p*: .01; Mann–Whitney *p*: .01) with a clear dichotomy at 15% OH, an established cutoff point for future adverse events [12,22–24]. This is also illustrated by Figure 1’s scatter plots, demonstrating the medians and standard deviation intervals for both groups. Our data were also examined in a categorical fashion relative to the 15% OH cutoff (Table 2). The majority of CEF patients were in the >15% OH category with the two-tailed Fisher’s exact test of association significant (*p*: .02). The unadjusted odds ratio of those with a CEF being in fluid excess was 5.3 (95% CI: 1.3–21.7; *p*: .01; the Number Needed to Harm was 4).

**Table 1.** Demographics and results comparing hemodialysis patients with and without a clinically enlarged fistula.

	Clinically enlarged fistulas (*n*: 13)	Control (*n*: 52)	*p*
Gender (M:F)	84.6%	51.9%	.03
Age (years)	51.1 ± 14.7	59.0 ± 14.7	.09
Dialysis vintage (years)	5.3 ± 2.4	4.8 ± 3.8	.62
Diabetes (%)	8	23	.22
Residual urine volume (mL/day)	274 ± 218	169 ± 295	.23
Number of antihypertensive medications	1.77 ± 1.4	0.80 ± 0.8	.002
Diuretics use (%)	15.3	9.6	.56
Body mass index (kg/m^2^)	25.1 ± 6.5	26.7 ± 5.7	.40
Systolic blood pressure (mmHg)	156 ± 24.5	141 ± 20.8	.003
Diastolic blood pressure (mmHg)	91 ± 10.9	78 ± 9.7	<.0001
OH (L)	4.2 ± 3.2	2.8 ± 1.6	.03
OH%	20.2 ± 7.4	14.4 ± 7.1	.01
EC (L)	19.7 ± 6.0	20.2 ± 18.7	.92

OH: overhydration: OH%: percent overhydration: EC: extracellular fluid volume.

When continuous data are shown, the means ± standard deviation is displayed.

**Table 2.** Results comparing those with and without a CEF in a 2 × 2 table for Fisher’s exact test (*p*: .02).

Data analyzed	CEF+	CEF–	Total
OH+	10	20	30
OH–	3	32	35
Total	13	52	65

CEF: clinically enlarged (arteriovenous) fistula; OH: overhydration; overhydration (OH) was dichotomized at a 15% excess of extracellular fluid; CEF+: patients with a CEF; CEF–: patients without a CEF; OH+: overhydrated; OH–: not overhydrated.

## Discussion

The presence of a high-flow hemodialysis fistula is associated with multiple adverse outcomes. These include steal syndrome [25], exotic complications such as hemothorax [26] or pulmonary embolism [27], and the impairment of cardiac function with high-output heart failure resulting from a large proportion a large proportion of the cardiac output recirculated through the enlarged fistulas [22,28,29]. While our working definition of CEF in our study was not harmonized to the diagnosis of ultrasonography-defined megafistula with high overall flow nor did we perform the Nicoladoni-Branham maneuver, our study entertains yet another striking possibility contributing to enlargement of arteriovenous accesses; the one of chronic salt-water overload. Only recently, exact technology with bioimpedance analysis become available for the daily routine care of ESRD patients, to offer reliable and repeated measures of volume status with a minimized burden and cost for the dialysis networks [9,14,15]. Our study shows that patients with a CEF have excess fluid of about 1.4 L over the control group. While this quantity does seem small, it is important to note that 10 out of 13 patients with a CEF had severe pre-dialysis volume overload as compared to only 20 of the 52 patients without CEF. We deemed fluid overload as ‘severe’ when the degree of overhydration (OH%) was greater than 15% [12,19]. Expressing fluid overload in liters is important to help individual patients understand target weight goals during renal replacement therapy, whereas expressing OH in percent is important for prognosis and securing comparability among clinical studies. As Wizemann et al. [12] showed, hemodialysis patients’ mortality is strongly affected when this threshold is met or exceeded. Interestingly, Wizemann’s study also showed that the presence of hypertension may be somewhat protective (hazard ratio: 0.98, *p*: .01) from mortality. Thus, we used the 15% OH cutoff to define the tolerance for fluid excess. The sustained fluid excess was not related to interdialytic weight gain – it hardly ever is – but rather to the inadequate determination and failure to reach the estimated dry weight [15]. In this particular cohort, we routinely observed that patients with CEF have steadfastly refused physician-recommended dry weight reduction and insisted that their weight be maintained at the level they deemed appropriate for themselves. There could be many reasons why these patients refused dry weight reduction; one possible explanation is the relatively long vintage (5.3 years) with the patients’ sense of empowerment of ‘knowing it better’. Also, the long vintage, could have contributed merely to the development of CEF, given so many cannulations over the years. In the past, we have demonstrated that one possible reason for fluid overload in dialysis patients is due to the difficulties reaching target net ultrafiltration goals while taking multiple anti-hypertensive drug medications [16]. In our current study, the linear regression analysis coefficient was 0.673 (*p* < .0001), between the number of antihypertensive medications and the amount of excess fluid (in liters), as measured by bioimpedance. Further, there was a strong correlation (*r* = 0.54, *p* < .0001) between the number of antihypertensive medications and the degree of fluid overload. Antihypertensive medications pose a risk of intradialytic hypotension, cramping and does appear to interfere with adequate ultrafiltration [9,16,30,31]. Indeed, this CEF patient cohort was taking a significantly larger number of antihypertensive medications (1.7 vs. 0.8; *p*: .002) and they were – perhaps consequently – more fluid overloaded. The use of diuretics did not appear to be protective. Our study reinforces the importance of validated technologies in determining the optimal target weight in ESRD patients as they are at risk for both hypo- and hypervolemia during treatment, if to rely on clinical assessment alone [9,16,30,31].

The question of interest raised by our study is whether this measured fluid excess is simply a measurement capacitance (the cumulative volume of enlarged fistulas) or the cause or exacerbating factor for the development of the CEF. The fact that these patients were taking more antihypertensive medications indicates a strong possibility of long-standing fluid overload, in keeping with the slow speed of enlarged fistula formation. As shown in Table 1, prescribing more anti-hypertensive drugs may have been the rule for these subjects, further aggravating the tendency to both fluid overload and potential CEF formation. While conventional treatment for these high-flow accesses is inflow restriction [6,32], the treatment may also involve optimizing fluid status.

Our study has several limitations. As CEF formation is a rare event, we compensated for the low number of index patients by ensuring that the comparator control group had plenty of patients, four times as many as the group with the condition. The lack of ultrasound-measured fistula flow determination was an obvious limitation of our study. We also did not perform cardiac ultrasound investigations as the focus of the study was fluid excess and its association with the presence of fistula enlargement, even though persisting fluid excess does have cardiac consequences as well. We have no information on previous endovascular interventions. Most importantly, the largest limitation of this study was the case-control design and observational nature of the project; therefore, a definitive conclusion cannot be drawn at this time. On the other hand, it would have been ethically impossible to perform a prospective study of fluid overloaded patients. Future studies need to explore this relationship further and extend these investigations to include measurements of cardiovascular health and cardiac output among these patients.

## Conclusions

While the observational nature of this study does not permit to imply causality, we observed a striking association between CEF formation and OH in a prevalent cohort of otherwise stable ESRD patients. This novel association may represent yet another consequence of poorly controlled volume status in ESRD and additional studies needed to provide independent verification. Future trials need to consider the presence of fistula enlargement when prospectively evaluating bioimpedance analysis and volume status among those being started on or receiving maintenance dialysis.
